# Impact of Particle Shape and Surface Group on Membrane Fouling

**DOI:** 10.3390/membranes12040403

**Published:** 2022-04-04

**Authors:** Melike Begum Tanis-Kanbur, Navin Raj Tamilselvam, Hsiao Yu Lai, Jia Wei Chew

**Affiliations:** 1 School of Chemical and Biomedical Engineering, Nanyang Technological University, Singapore 637459, Singapore; melike.btk@ntu.edu.sg (M.B.T.-K.); navinraj002@e.ntu.edu.sg (N.R.T.); hsiaoyu.lai@ntu.edu.sg (H.Y.L.); 2 Singapore Membrane Technology Centre, Nanyang Environment and Water Research Institute, Nanyang Technological University, Singapore 637141, Singapore

**Keywords:** microfiltration, microplastics, membrane fouling, microparticles, interaction energy, shear-induced diffusion, critical flux

## Abstract

Membrane fouling remains one of the most critical drawbacks in membrane filtration processes. Although the effect of various operating parameters—such as flow velocity, concentration, and foulant size—are well-studied, the impact of particle shape is not well understood. To bridge this gap, this study investigated the effect of polystyrene particle sphericity (sphere, peanut and pear) on external membrane fouling, along with the effect of particle charge (unmodified, carboxylated, and aminated). The results indicate that the non-spherical particles produce higher critical fluxes than the spherical particles (i.e., respectively 24% and 13% higher for peanut and pear), which is caused by the looser packing in the cake due to the varied particle orientations. Although higher crossflow velocities diminished the differences in the critical flux values among the particles of different surface charges, the differences among the particle shapes remained distinct. In dead-end filtration, non-spherical particles also produced lower flux declines. The shear-induced diffusion model predicts all five particle types well. The Derjaguin-Landau-Verwey-Overbeek (DLVO) and extended DLVO (XDLVO) models were used to quantify the interaction energies, and the latter agreed with the relative critical flux trends of all of the PS particles. As for the flux decline trends, both the DLVO and XDLVO results are in good agreement.

## 1. Introduction

Membrane-based separation is widely used in water/wastewater treatment, food processing, and bioprocesses, thanks to its relatively affordable and easier operations compared to traditional techniques [1,2,3,4]. Unfortunately, membrane fouling, which causes a decrease in permeate flux over time [5], remains the biggest drawback [6,7,8]. Fane et al. [9] classified the fouling phenomenon into three categories, namely the closure of pores, pore plugging, and cake layer formation, among which cake formation is dominant until the end of the filtration [10]. Crossflow microfiltration, in which the feed is pumped across the membrane surface to confer a tangential shear to mitigate fouling, is a common practice [11]. The extent of membrane fouling or particulate deposition onto the membrane surface depends on several parameters, such as the crossflow velocity, feed concentration, particle type and size [12,13,14], and membrane surface characteristics [15], which have unsurprisingly been studied well. However, although the foulants are likely not perfectly spherical, the effect of particle non-sphericity on membrane fouling remains poorly understood.

Connell et al. [16] found that particle shape affected the flux trends, with irregularly shaped particles achieving less fouling, which has been tied to spherical particles forming more uniform cakes with greater resistance. Smidova et al. [17] also reported that particle shape affected flux trends and fouling. Wang et al. [18] reported that particles of different sphericities exhibited different particle velocity distributions and different orientation velocities at the same liquid flow rate. Abdelrasoul et al. [19] developed a mathematical model describing membrane fouling by polydisperse latex particles with different shapes, and noted that membranes with uniform pore sizes and straight pores are highly affected by particle shape and sphericity parameters.

Apart from the membrane-filtration field, particle sphericity effects and their corresponding consequences have been reported in terms of different segregation extents [20], different energy requirements for fluidization [21,22], different fluidizing qualities [23], different contact forces and particle velocities [24], and different rotation tendencies [25]. A systematic, targeted study on the effect of particle shape on membrane fouling would be useful.

Because the research gap with respect to the impact of particle shape on membrane fouling is clear, this study focused on understanding and comparing the effects of particle charge and shape during the microfiltration of micron-sized polystyrene foulant particles. Five particle types were investigated: three sphere-shaped ones of different surface charges (unmodified, aminated, and carboxylated), as well as two non-spherical ones (peanut and pear). The Direct Observation Through the Membrane (DOTM) technique was used to obtain images of the feed-membrane interface in real time, and to determine the critical flux. The critical flux results were also compared with those predicted by the Shear-induced Diffusion (SID) model. In addition to the crossflow filtration via the DOTM technique, a dead-end filtration setup was employed in order to understand the impact of crossflow on fouling by different particle types. Besides this, the Derjaguin-Landau-Verwey-Overbeek (DLVO) and extended DLVO (XDLVO) models were used to calculate foulant–membrane interactions.

## 2. Theory

### 2.1. Shear-Induced Diffusion (SID) Model

In the crossflow implemented in membrane-filtration modules to mitigate membrane fouling, the velocity fields cause particle self-diffusion in the shear flow, which is described by shear-induced hydrodynamic diffusion models [26]. Li et al. [27] reported that, for smaller latex particles of 3 µm, similar to those in this study, the shear-induced diffusion (SID) model has to be modified to the following:

(1) Jcrit=0.807(γ0D2L)13ln(ϕwϕb)

where $J_{crit}$ is the critical flux (L/m^2^h), $\gamma_{0}$ represents the shear rate at the membrane surface (s^−1^, calculated by dividing the crossflow velocity by feed channel height), D represents the diffusivity (m^2^/s, calculated using $\frac{D}{a^{2}\gamma_{0}}=$ $C_{d}\phi_{w}$, where $C_{d}=\frac{J/a^{4/3}\;}{\gamma_{0}}$ and *a* is the foulant diameter [27]), $\phi_{w}$ represents the particle volume fraction at the surface of the membrane (set at 0.13 based on a membrane area coverage of 20% [27]), $\phi_{b}$ represents the particle volume fraction in the bulk feed solution, and *L* represents the membrane length (m).

### 2.2. DLVO and Extended DLVO (XDLVO) Models

According to the Derjaguin-Landau-Verwey-Overbeek (DLVO) theory, the total interfacial interaction is expressed as the summation of Lifshitz–van der Waals (LW) and electrostatic double-layer (EL) terms [28,29], given in Equation (2):

(2) $$U_{mlc}^{DLVO}=U_{mlc}^{LW}+U_{mlc}^{EL}$$

where U represents the interaction energy, and the subscripts m, l, and c indicate the membrane, liquid environment, and colloid, respectively. The extended DLVO theory (XDLVO) includes an additional Lewis acid–base (AB) component [30], which improves the predictability for membrane fouling in aqueous media [30,31]:

(3) $$U_{mlc}^{XDLVO}=U_{mlc}^{LW}+U_{mlc}^{EL}+U_{mlc}^{AB}$$

#### 2.2.1. Lifshitz–van der Waals (LW) Interaction

The LW interaction energy is the free energy of adhesion per unit area ($∆G_{yo}^{LW}$) between two surfaces that accounts for the non-polar interaction:

(4) $$∆G_{y_{o}}^{LW}=2\left(\sqrt{\gamma_{l}^{LW}}-\sqrt{\gamma_{m}^{LW}}\right)\left(\sqrt{\gamma_{c}^{LW}}-\sqrt{\gamma_{l}^{LW}}\right)$$

where $y_{0}$ is the minimum equilibrium cut-off distance between the two surfaces, and is assigned a value of 0.158 nm [30], and γ symbolizes the surface tension.

The LW interaction energy as a function of the separation distance (h) between a colloid and a membrane can be calculated from

(5) $$U_{mlc}^{LW}\left(h\right)=2\pi ∆G_{y_{0}}^{LW}\frac{y_{0}^{2}a_{c}}{h}$$

where $a_{c}$ is the radius of the colloid.

#### 2.2.2. Electrostatic Double-Layer (EL) Interaction

The EL interaction is related to the surface zeta potentials, and the free energy per unit area ($∆G_{yo}^{EL})$ between two surfaces is expressed as

(6) $$∆G_{y0}^{EL}=\frac{\varepsilon_{0}\varepsilon_{r}\kappa }{2}\left(\zeta_{m}^{2}+\zeta_{c}^{2}\right)\left(1-coth\left(\kappa y\right)+\frac{2\zeta_{m}\zeta_{c}}{\left(\zeta_{m}^{2}+\zeta_{c}^{2}\right)}csch\left(\kappa y\right)\right)$$

where $\zeta_{m}$ and $\zeta_{c}$ are the zeta potentials of, respectively, the membrane and the colloid, $\varepsilon_{0}$ is the vacuum permittivity (8.854 × 10^−12^ C/Vm), $\varepsilon_{r}$ is the dielectric constant of the liquid medium (80 for DI Water [32]), and *κ* is the inverse Debye screening length. As for *κ*, it was calculated as follows:

(7) $$\kappa^{-1}=\sqrt{\frac{D\varepsilon_{0}\varepsilon_{r}}{\sigma }}$$

where D is the diffusion coefficient (10^−9^ m^2^/s) [33] and σ is the electrical conductivity of the liquid medium. Particularly, σ was measured to be 1.162 µS/cm for DI Water using a conductivity meter (SevenCompact, Mettler Toledo).

The EL interaction energy as a function of the separation distance (h) between a colloid and membrane can be calculated from

(8) $$U_{mlc}^{EL}\left(h\right)=\pi \varepsilon_{0}\varepsilon_{r}a_{c}\left[2\zeta_{m}\zeta_{c}\ln\left(\frac{1+e^{-\kappa h}}{1-e^{-\kappa h}}\right)+\left(\zeta_{m}^{2}+\zeta_{c}^{2}\right)\ln\left(1-e^{-2\kappa h}\right)\right]$$

#### 2.2.3. Lewis Acid–Base (AB) Interaction

The AB interaction denotes the polar interaction represented by the electron acceptor $\left(\gamma^{+}\right)$ and electron donor $\left(\gamma^{-}\right)$ parameters of the surface tension. The free energy of adhesion per unit area ($∆G_{y0}^{AB}$) between two surfaces can be expressed as follows:

(9) $$∆G_{y0}^{AB}=2\sqrt{\gamma_{l}^{+}}\left(\sqrt{\gamma_{m}^{-}}+\sqrt{\gamma_{c}^{-}}-\sqrt{\gamma_{l}^{-}}\right)+2\sqrt{\gamma_{l}^{-}}\left(\sqrt{\gamma_{m}^{+}}+\sqrt{\gamma_{c}^{+}}-\sqrt{\gamma_{l}^{+}}\right)-2\left(\sqrt{\gamma_{m}^{+}\gamma_{c}^{-}}+\sqrt{\gamma_{m}^{-}\gamma_{c}^{+}}\right)$$

The AB interaction energy as a function of the separation distance (h) between a colloid and membrane is given as

(10) $$U_{mlc}^{AB}\left(h\right)=2\pi a_{c}\lambda ∆G_{y_{0}}^{AB}exp\left[\frac{y_{0}-h}{\lambda }\right]$$

where λ is the characteristic decay length of the AB interaction in water, and the commonly used value for aqueous systems is 0.6 nm [34].

The overall surface tension ($\gamma^{TOT}$) is the summation of the non-polar (LW) and polar (AB) components:

(11) $$\gamma^{TOT}=\gamma^{LW}+\gamma^{AB}$$

where the polar surface tension component $\gamma^{AB}$ is a function of the electron acceptor $\left(\gamma^{+}\right)$ and electron donor $\left(\gamma^{-}\right)$ parameters of the surface tension:

(12) $$\gamma^{AB}=2\sqrt{\gamma^{+}\gamma^{-}}$$

The surface energetic parameters for the alumina membrane, water, and polystyrene (PS) are listed in Table 1.

## 3. Materials and Methods

### 3.1. Chemicals and Reagents

**Figure 1:**
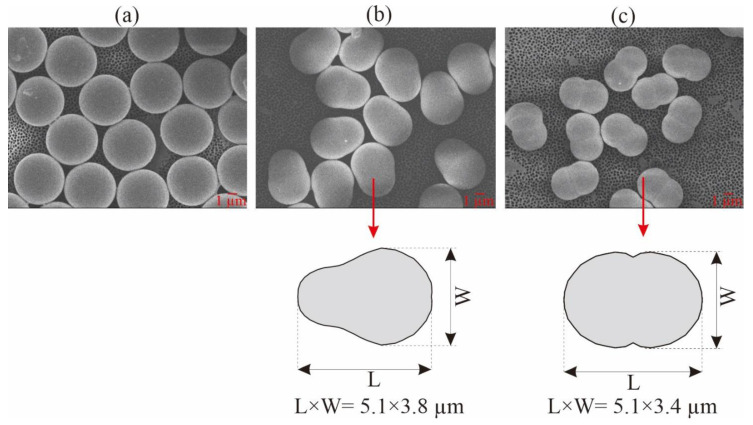
FESEM images of the PS microparticles: (**a**) spherical, (**b**) pear-shaped, and (**c**) peanut-shaped.

**Figure 2:**
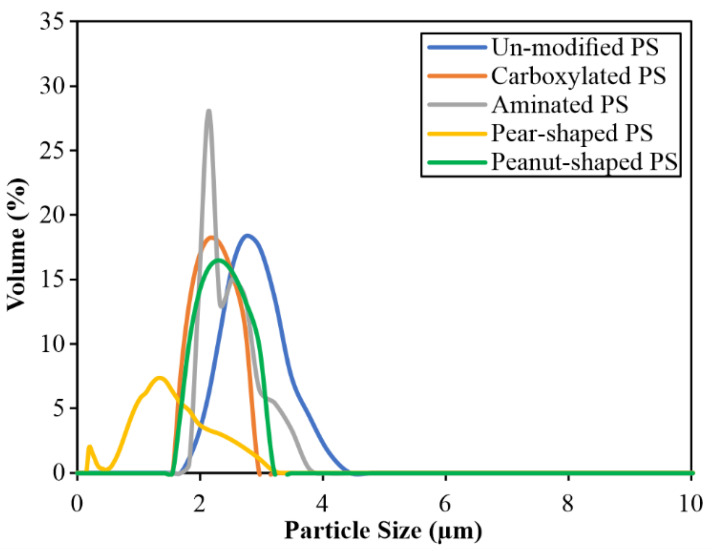
Particle size distributions (PSDs) of the spherical and non-spherical PS microparticles. Each PSD is the mean of three measurements.

The five different polystyrene (PS) microparticles (particle density = 1.05 g/mL) used in this study were purchased from Magsphere Inc. (Pasadena, CA, USA). Three were spherical (unmodified (PS005UM), carboxylated (CA005UM), and aminated (AM005UM)) and two were non-spherical (pear-shaped (PNT005UM) and peanut-shaped (PNT005UM)). Figure 1 shows the high-magnification field emission scanning electron microscope (FESEM; JEOL JSM-6701F; 5 kV accelerating voltage and high-vacuum (9.63 × 10^–5^ Pa) mode) images of the spherical, pear-shaped, and peanut-shaped PS microparticles. All of the FESEM samples were coated with platinum in order to avoid the charging effect during the imaging. The vendor-given dimensions (listed in Table 2) specified that the spherical particles had diameters of approximately 5 μm (Figure 1a), the pear-shaped ones had, respectively, a maximum length and width of 5.1 and 3.8 μm, and the peanut-shaped ones had, respectively, a maximum length and width of 5.1 and 3.4 μm. The particle dimensions were also measured via FESEM, and were found to be similar to the vendor-given values, as listed in Table 2. The particle size distributions and zeta potentials of the feed samples (i.e., either 50 mg/L or 25 mg/L of PS in DI water (Milli-Q DI Water Purification System (Merck-Millipore, Burlington, MA, USA)) were measured using the particle size analyzer (Litesizer 500, Anton Paar, Graz, Austria). The volume-based particle size distributions (PSDs) are presented in Figure 2, reflecting that the sizes were below 5 μm, with mean values between 1.1–2.8 µm (Table 2). While all of the particles investigated fell within a narrow size range, the width of the PSD of the pear-shaped PS was distinctly different, likely due to the varied orientations during the size analysis.

The membranes used in the experiments were inorganic, namely Anopore alumina (Anodisc, Whatman, Buckinghamshire, UK) with a diameter of 47 mm and a nominal pore size of 0.2 μm. The membrane was glued (Araldite) between two pieces of paper (55 mm by 135 mm) from which a square (27 × 27 mm) was cut out from the center in order to expose an active membrane area of 7.29 cm^2^.

### 3.2. Determining Particle Sphericity

The particle sphericity can be quantified using the following [37]:

(13) $$\varphi =\frac{\pi \cdot d_{eq}^{2}}{SA}$$

where $\pi \cdot d_{eq}^{2}$ is the surface area of the volume-equivalent sphere, and SA is the particle surface area. The unmodified, carboxylated, and aminated PS particles were spherical, as seen in Figure 1a, giving φ as 1.

Following the former studies that focused on the pear-like [38,39] and peanut-like [40,41] shapes in different applications, the sphericity values of the pear and peanut-shaped particles were calculated from Equation (13) as 81.8% and 51.6%, while the corresponding particle radii values are 1.90 and 1.89 µm, respectively.

### 3.3. Experimental Setups

**Figure 3:**
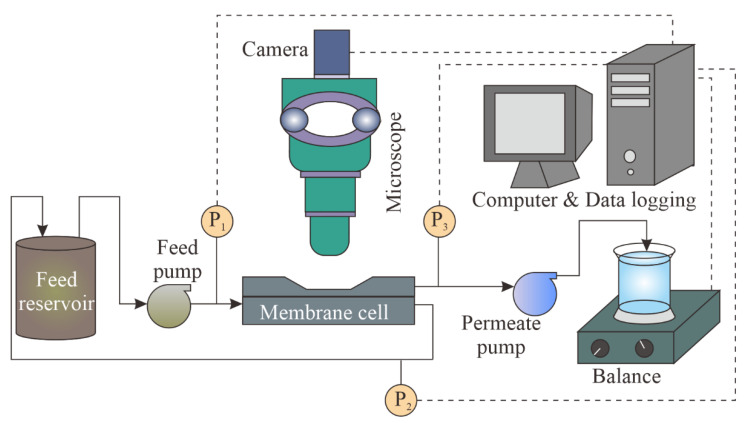
Schematic diagram of the Direct Observation Through the Membrane (DOTM) setup.

Figure 3 presents the schematic of the Direct Observation Through the Membrane (DOTM) setup, which involves a camera (Axiocam 105 Color, Zeiss, Oberkochen, Germany) coupled with a light microscope (Axio Imager.A2m, Zeiss, Oberkochen, Germany) to obtain a direct visual observation of the membrane fouling during the filtration. The DOTM technique has been used popularly for crossflow microfiltration applications involving a wide range of foulants, including oil emulsions [13,14], bacteria [42], algae [43], hematite flocs [44], particulate foulants [45], surfactants [13,14] and organic solvents [46]. In particular, the Anopore membrane becomes transparent when it is wet, which is required for the DOTM technique. In order to focus through the transparent membrane at the feed–membrane interface, the objective of the microscope was placed above the crossflow membrane module, which was made of acrylic to allow light transmission. The membrane module had dimensions of 105 mm in length, 35 mm in width, and 3 mm in height, with the feed and permeate channel heights being 2 mm and 1 mm, respectively. Additionally, the other system components were a gear pump (GJ-N25.PF/S.A, Micropump Inc., Vancouver, WA, USA) to control the feed flow (and thus to set the targeted crossflow velocity (CFV)); a peristaltic pump (Masterflex L/S 7519-20/85, Cole-Parmer, Vernon Hills, IL, USA) to control the permeate flow; three pressure transmitters (Transducer 206, Cole-Parmer, Vernon Hills, IL, USA) to measure the feed inlet pressure, feed outlet pressure, and permeate inlet pressure (and thus the transmembrane pressure, which is the difference between the feed and permeate pressures); a 600 mL glass beaker containing the feed solution, which was placed on a magnetic stirrer plate (MR Hei-Mix S, Heidolph, Schwabach, Germany); a 100 mL glass beaker containing the permeate placed on top of a balance (572, Kern, Balingen, Germany) to measure the permeate mass; and a computer to record the images via the microscope software (Zen 2.3 lite, Zeiss, Oberkochen, Germany), and also to log the pressure signals from the pressure transmitters and mass from the balance via LabVIEW (2014-64 bit). For each crossflow experiment, 500 mL of the feed solution with the desired PS concentration (i.e., 25 and 50 mg/L) was prepared, and the particles were kept homogenously suspended in DI water using a magnetic stirrer. For the feed solutions containing mixed PS microparticles, the composition was 50% by volume of each particle type, namely 50% unmodified and 50% pear-shaped, and 50% unmodified PS and 50% peanut-shaped PS.

**Figure 4:**
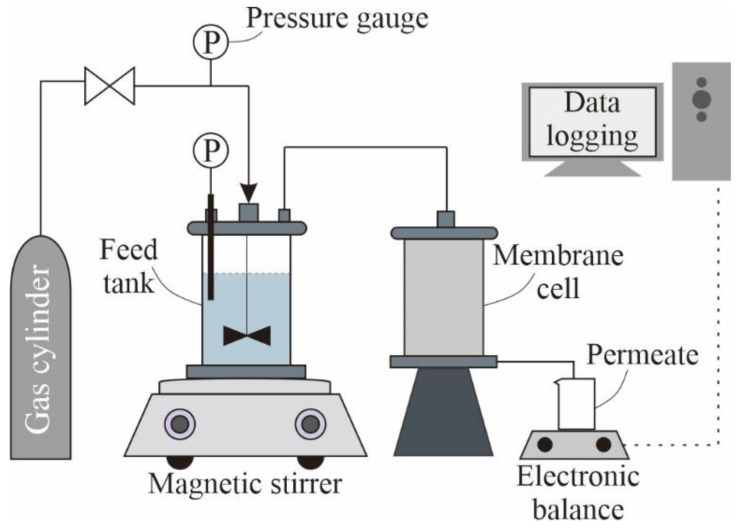
Simplified schematic of the dead-end filtration setup.

Dead-end filtration was also performed to investigate the effect of crossflow. The experimental setup is presented in Figure 4 [47]. The setup was made of stainless steel and included a membrane unit with an active membrane area of 1 cm^2^ and a feed tank with a volume of 1000 mL. The tubings were made of polytetrafluoroethylene (PTFE; Teflon, Masterflex, Cole-Parmer, Vernon Hills, IL, USA). A compressed air cylinder (Leeden, Singapore) was used to supply the necessary pressure to drive the filtration. The transmembrane pressure (TMP) was monitored based on the difference between the pressure gauge on the air cylinder and the PTFE-diaphragm pressure gauge (0 to 200 psi) in the membrane module. During the filtration, the permeate was collected in a glass container with a volume of 1000 mL, which was placed on an electronic balance (ML4002, Mettler Toledo, Columbus, OH, USA), and the mass was logged every 60 s via a piece of software (Balance Link, Mettler Toledo, Columbus, OH, USA). For each dead-end filtration experiment, 400 mL of feed was prepared with 5 mg/L PS.

### 3.4. Critical Flux Determination

The critical flux (*J_crit_*) is the flux above which membrane fouling onsets [48]. In this study, the critical flux values were identified by using the flux-stepping method [49]. The initial permeate flux was fixed at 5 L/m^2^h for 10 min, and after that, the flux was increased by 5 L/m^2^h every 10 min. At every permeate flux, an image was taken at the second minute, then another image was taken at the tenth minute. All of the images were analyzed using ImageJ to calculate the rate of change of the surface coverage (∆*C*/∆*t*):

(14) $$\frac{∆C}{∆t}=\frac{C_{10th\;minute}-C_{2nd\;minute}}{t_{10th\;minute}-t_{2nd\;minute}}$$

**Figure 5:**
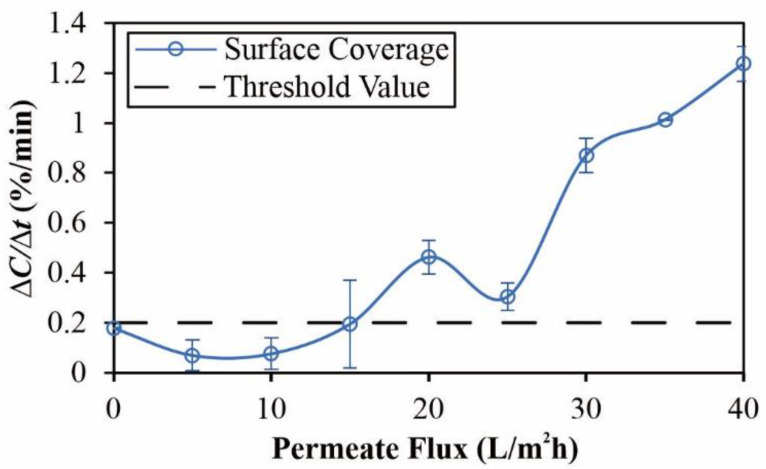
Plot of ∆C/∆t versus the permeate flux for 50 mg/L of aminated PS at CFV = 0.1 m/s. In this case, the average critical flux was determined to be 14.0 L/m^2^h. Each error bar represents the range of data obtained for at least two repeats.

Figure 5 presents a representative rate of change of concentration $\left(\frac{∆C}{∆t}\right)$ versus the permeate flux plot. The critical flux (*J_crit_*) was determined as the permeate flux at which $\frac{∆C}{∆t}$ exceeded a pre-determined threshold, which was determined as 0.2%/min, as per previous studies [13,14,45]. The reported *J_crit_* values were based on duplicate or triplicate experiments, with error bars reflecting the range of data. Three crossflow velocities (CFV) in the range of 0.1–0.4 m/s were investigated, which correspond to the Reynolds number (calculated by $\frac{\rho \times D_{eq}\times \mathrm{CFV}}{\mu }$, where ρ and μ are the density and viscosity of the feed, and $D_{eq}=\frac{2\times H\times W}{H+W}$ where *H* and *W* are, respectively, the height and width of the feed channel) in the range of 419 and 1677 [13].

## 4. Results and Discussion

### 4.1. Crossflow Studies

**Figure 6:**
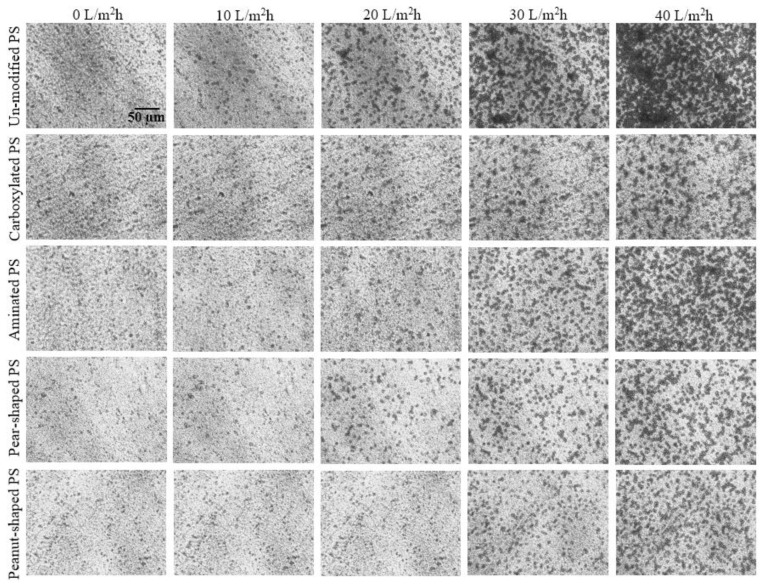
Surface coverage images via the DOTM technique for different PS microparticles (unmodified, carboxylated, aminated, pear-shaped, and peanut-shaped) and permeate fluxes (0–40 L/m^2^h) at CFV = 0.1 m/s. All of the feeds had the same PS particle concentration of 50 mg/L. The images were taken at the end of the 10th minute of each permeate flux step.

In order to assess the effect of surface charge and particle shape on membrane fouling, Figure 6 presents the DOTM images at five fluxes (in the range of 0 to 40 L/m^2^h) for the five PS particle types. Specifically, the increase in dark pixels at the higher fluxes reflects the increase in particle deposition onto the membrane. Because the DOTM images are two-dimensional, it is not possible to infer the cake layer thickness, but the images clearly show the extent of the particle deposition. Among the spherical particles, the unmodified particles exhibited the highest fouling, followed by aminated and then carboxylated particles. Comparatively, the non-spherical particles (i.e., pear-shaped and peanut-shaped) produced less fouling than the spherical ones.

**Figure 7:**
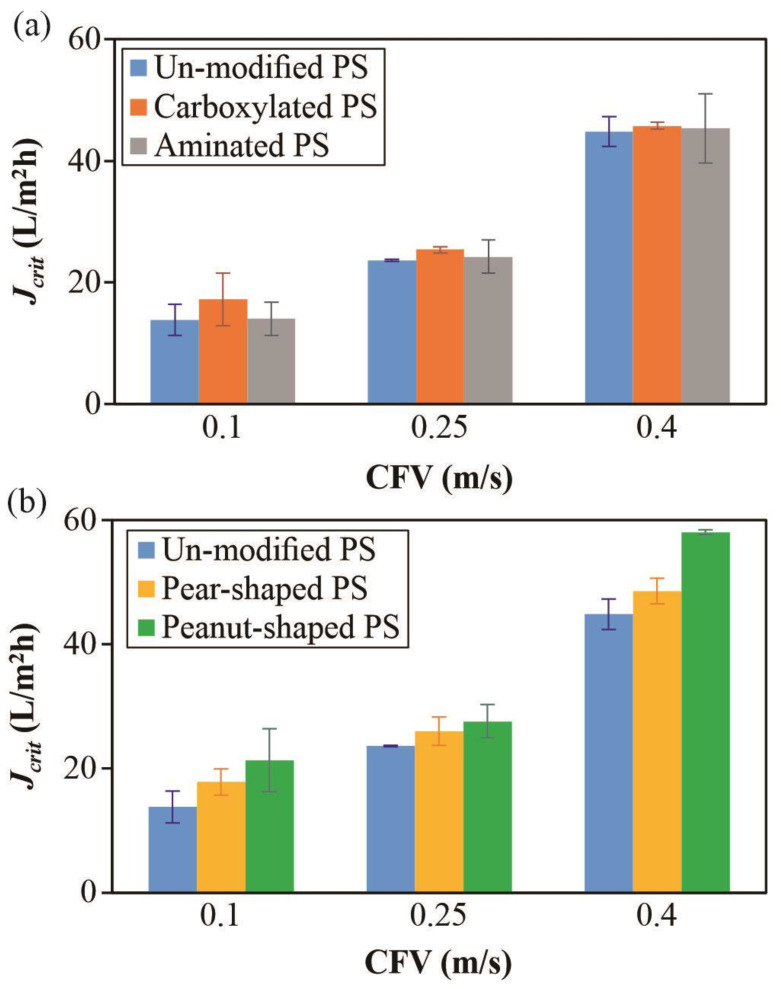
Critical flux (*J_crit_*) values of 50 mg/L of (**a**) PS microparticles with different surface groups, and (**b**) spherical and non-spherical PS microparticles at CFV values between 0.1 and 0.4 m/s. The error bars represent the range of *J_crit_* values from repeated experiments.

Figure 7a presents the relative critical flux (*J_crit_*) trends of the three spherical particles with different surface charges. The most significant difference among the particle types was at the lowest CFV, which agreed with an earlier study indicating that hydrodynamic effects diminish surface energy effects at higher CFVs [45]. That is, the increase in CFV makes the hydrodynamic effects (e.g., particle back transport) more notable, such that the critical flux difference for the samples with different surface charges is decreased by increasing CFV. The trends also show that the *J_crit_* values were not affected by the zeta potential. Although the carboxylated PS had a less negative zeta potential than the unmodified ones, the *J_crit_* values were higher, which can be linked to the surface modifications that confer increased hydrophilicity [50]. The relative trends agree with both the external [51] and internal [52] fouling trends reported by earlier studies that used Optical Coherence Tomography (OCT) for dead-end filtration. Figure 7b presents the relative critical flux (*J_crit_*) trends of the unmodified spherical and two non-spherical particles, which had more similar zeta potentials (Table 2), at different CFV values. Expectedly, all of the *J_crit_* values increased with increasing CFV. Irrespective of CFV, the peanut-shaped PS had the highest *J_crit_*, followed by the pear-shaped then the unmodified (spherical) ones. Specifically, the pear- and peanut-shaped particles had *J_crit_* values which were, respectively, 13% and 24% higher than the spherical ones. This indicates that spherical particles tend to exhibit more extensive fouling. This agrees with an earlier study indicating that highly irregularly shaped particles had higher flux trends than the spherical ones, which was tied to sphere-shaped particles depositing closer to form denser cakes [16]. Compared to Figure 7a, it is evident that higher CFV values did not diminish the differences in the *J_crit_* values among the particle types in Figure 7b. Other than packing effects, another feature of non-spherical particles in crossflow is the rotational effects. Higher CFV values accentuate the rotation [18], resulting in more significantly different *J_crit_* values at the highest CFV of 0.4 m/s.

**Figure 8:**
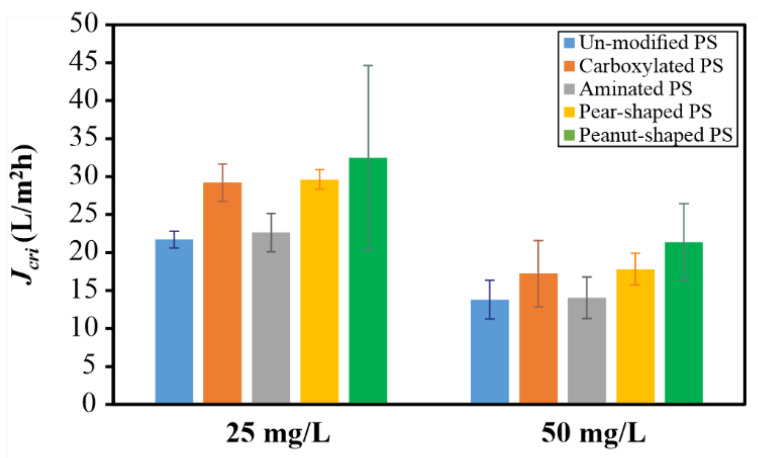
Effect of concentration (25 mg/L versus 50 mg/L) on the critical flux (*J_crit_*) at a CFV of 0.1 m/s. The error bars represent the range of *J_crit_* values from repeated experiments.

**Figure 9:**
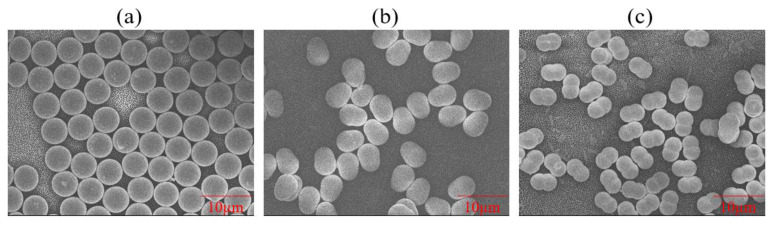
FESEM images of the fouled membranes with differently shaped PS microparticles after crossflow filtration via the DOTM technique: (**a**) unmodified PS, (**b**) pear-shaped PS, and (**c**) peanut-shaped PS. Permeate flux = 40 L/m^2^h. The images were taken at the end of the 10th minute. CFV = 0.1 m/s, and PS concentration = 50 mg/L.

In Figure 8, the *J_crit_* values are displayed for the five particle types at two different concentrations, namely 25 mg/L and 50 mg/L, at a CFV of 0.1 m/s. As expected, an increase in concentration decreased the *J_crit_* values. The relative trends among the different particle types remained consistent, suggesting that CFV had a greater effect than concentration on the distinct trends in the ranges considered. Apart from the DOTM images taken in real-time (Figure 6), FESEM images were taken at the end of the filtration experiments in order to better visualize the deposition patterns as shown in Figure 9. Clearly, the spherical particles packed closer, whereas the non-spherical ones packed relatively more loosely due to the varied orientations associated with the non-sphericity. This emphasizes the need to consider particle shape effects in membrane fouling models due to the orientation and packing effects.

**Figure 10:**
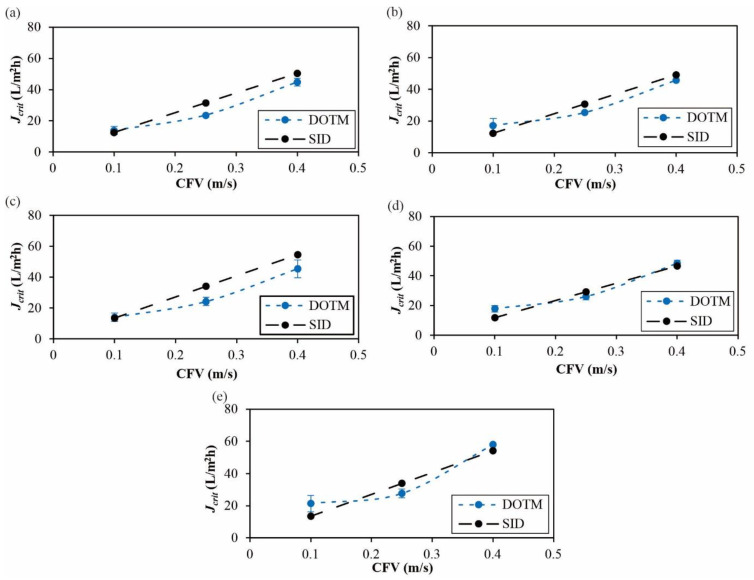
Comparison of the critical flux and SID model trends of the PS particles: (**a**) unmodified, (**b**) carboxylated, (**c**) aminated, (**d**) pear-shaped, and (**e**) peanut-shaped.

Furthermore, the *J_crit_* trends obtained by the DOTM agree with the SID model (Equation (1)) for all of the particle types (unmodified, carboxylated, aminated, pear-shaped, and peanut-shaped PS), as shown in Figure 10. This appears to suggest that the SID model is able to account for the differences caused by particles of different shapes and charges. Li et al. [27] were able to use the same model for both 3 µm latex and 4 µm algae particles, affirming the versatility of the model.

**Figure A1:**
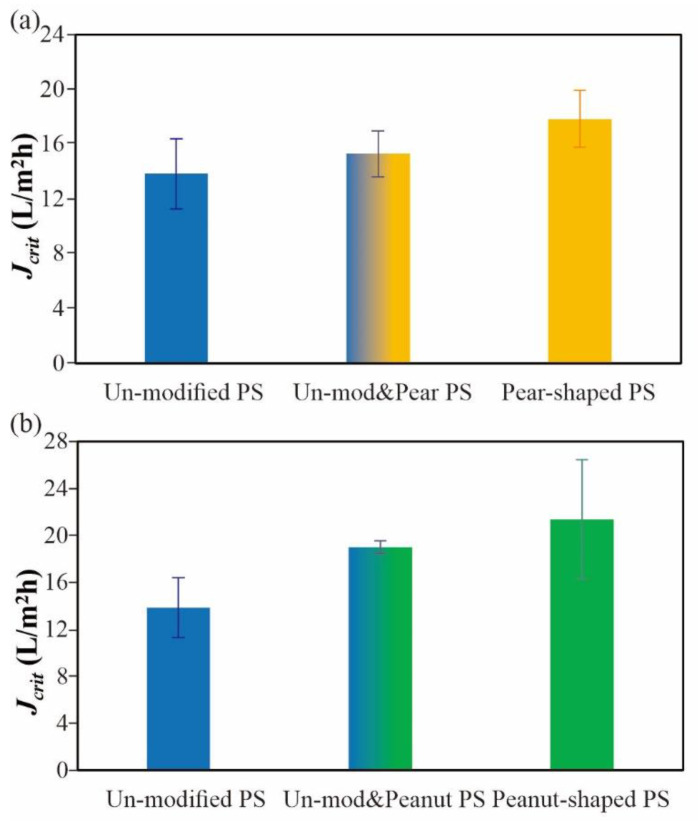
Critical flux (*J_crit_*) values at CFV of 0.1 m/s of 50 mg/L of mixtures of (**a**) 50 vol% of un-modified & 50 vol% of pear-shaped PS; and (**b**) 50 vol% of un-modified & 50 vol% of peanut-shaped PS. Error bars represent the ranges of *J_crit_* values from repeated experiments.

**Figure A2:**
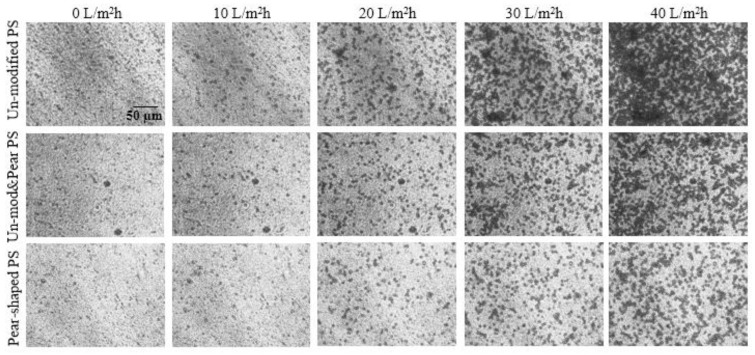
Surface coverage images via DOTM technique for different PS microparticles (namely, un-modified, 50% un-modified & 50% pear-shaped mixture, and pear-shaped) and permeate fluxes (0–40 L/m^2^h) at CFV = 0.1 m/s. All feeds had the same total PS particle concentration of 50 mg/L. The images were taken at the end of the 10th minute of each permeate flux step.

**Figure A3:**
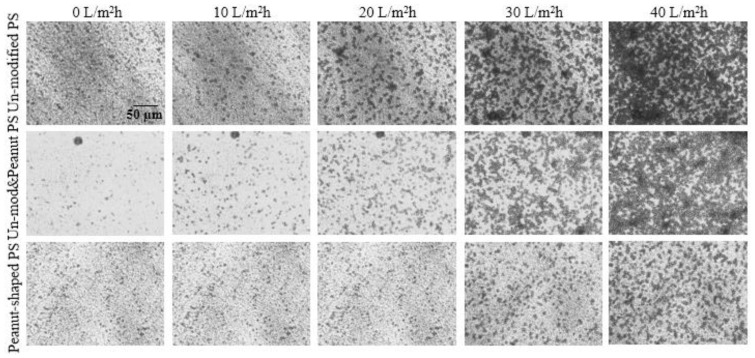
Surface coverage images via DOTM technique for different PS microparticles (namely, un-modified, 50% un-modified & 50% peanut-shaped mixture, and peanut-shaped) and permeate fluxes (0–40 L/m^2^h) at CFV = 0.1 m/s. All feeds had the same total PS particle concentration of 50 mg/L. The images were taken at the end of the 10th minute of each permeate flux step.

**Figure A4:**
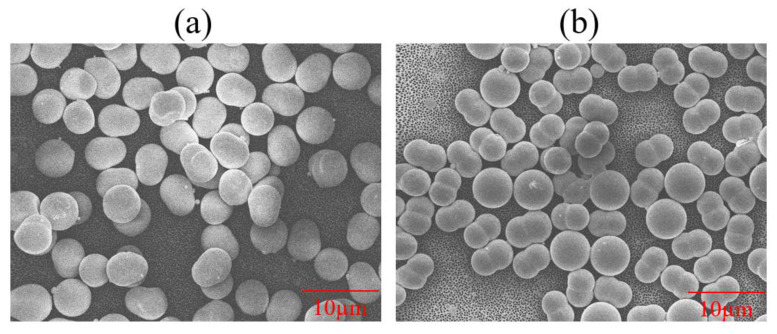
FESEM images of the fouled membranes with mixed PS microparticles after crossflow filtration via DOTM technique: (**a**) 50% un-modified PS & 50% pear-shaped PS mixture, and (**b**) 50% un-modified PS & 50% peanut-shaped PS mixture. (Permeate flux = 40 L/m^2^h and CFV = 0.1 m/s, total PS concentration = 50 mg/L).

Because practical feeds are made up of mixed particle types, the effect of mixtures was also investigated. Figure A1 presents the *J_crit_* values at the CFV value of 0.1 m/s of the mixtures (50 vol% unmodified–50 vol% pear-shaped PS, and 50 vol% unmodified–50 vol% peanut-shaped PS) vis-à-vis that of the constituents. Expectedly, the *J_crit_* values of the mixtures lie in between those of the constituents. The corresponding DOTM images are presented in Figure A2 and Figure A3. The FESEM images in Figure A4 show that the packing is increased compared to the non-spherical particles alone and decreased compared to the spherical particles alone.

### 4.2. Dead-End Studies

**Figure 11:**
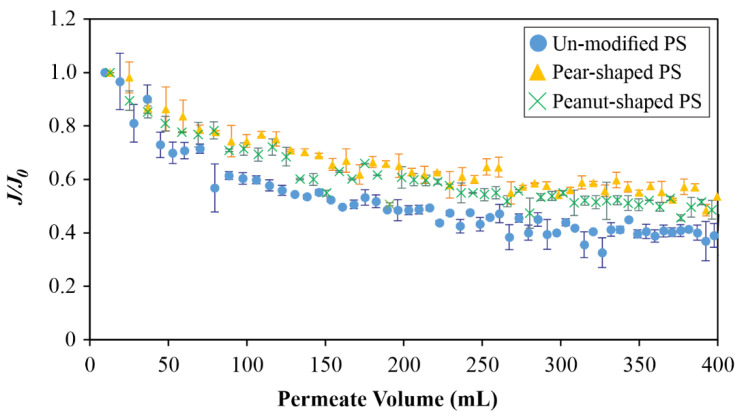
Normalized flux with respect to the permeate volume for feeds containing spherical and non-spherical PS microparticles. The concentration was 5 mg/L, and the TMP was 0.5 bar.

Figure 11 presents the normalized flux (*J/J_o_*) decline trends with respect to the permeate volume for 5 mg/L of three PS particle types (unmodified, pear-shaped, and peanut-shaped) at a fixed TMP of 0.5 bar. Clearly, the unmodified PS gave the greatest flux decline, while the pear- and peanut-shaped ones gave similarly slower declines. The relative flux decline trends disagree somewhat with the relative *J_crit_* trends in Figure 7b and Figure 8, specifically in that the peanut-shaped PS gave distinctly higher *J_crit_* values than the pear-shaped ones. The discrepancy can be linked to the particle orientations of such non-spherical particles in the presence and absence of crossflow. Connell et al. [16] reported that irregular shapes were further apart from one another, and gave a greater cake voidage that mitigated membrane fouling in crossflow filtration.

### 4.3. Interaction Energy

**Figure 12:**
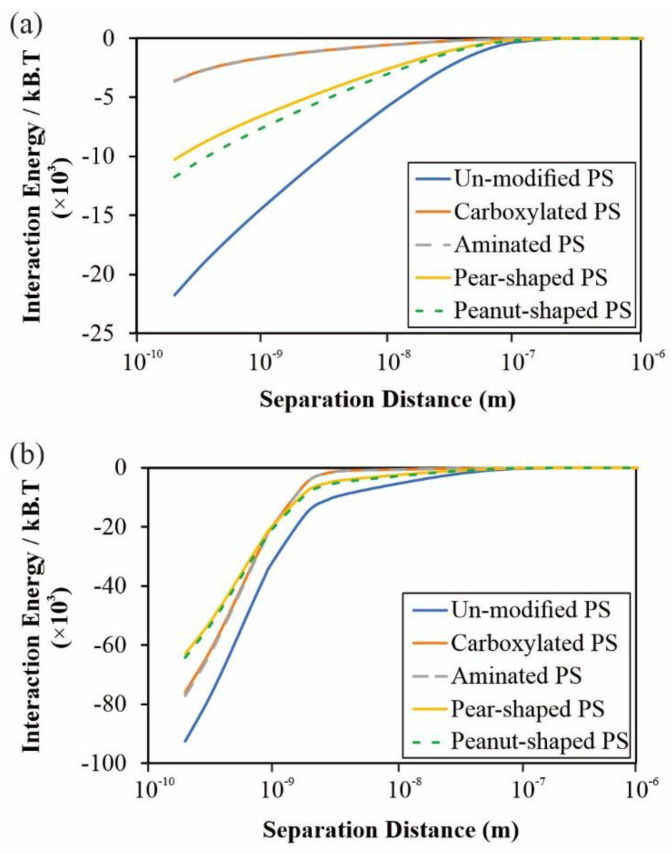
Interfacial PS–membrane interaction energy analysis via (**a**) DLVO and (**b**) XDLVO.

Interfacial interaction energy analysis between the membrane and particles was performed. Figure 12 presents the DLVO and XDLVO calculations as a function of the separation distance. For DLVO (Figure 12a), the most attractive energy is exhibited by the unmodified PS, followed by peanut-shaped, pear-shaped, then carboxylated and aminated particles. Because higher interaction energy between the membrane and the foulant is related to intensive fouling, the *J_crit_* trends of spherical particles (unmodified, carboxylated, and aminated PS) in the DOTM experiments (Figure 7 and Figure 8) are in good agreement with the DLVO results. However, the DLVO trends of the non-spherical particles do not agree with the *J_crit_* trends, as the interaction energies of the pear- and peanut-shaped particles are in between those of the unmodified PS and the aminated PS. As for XDLVO (Figure 12b), the relative trends differ from those of DLVO, specifically, with unmodified particles persisting to be the most attractive, but the carboxylated and aminated PS were more attractive than the pear- and peanut-shaped PS. Unlike the DLVO calculations, the XDLVO calculations (Figure 12b) were in much better agreement with the *J_crit_* trends of both the spherical and non-spherical PS particles. As the unmodified PS exhibited the highest interaction energy, both the DLVO and XDLVO results are in good agreement with the flux decline trends presented in Figure 11, in which unmodified PS produced a faster flux decline than the pear-shaped and peanut-shaped PS particles.

## 5. Conclusions

This study investigated the impact of polystyrene (PS) particle surface charge (unmodified, aminated and carboxylated) and shape (sphere-, pear- and peanut-shaped) on membrane fouling during crossflow and dead-end microfiltration. The Direct Observation Through the Membrane (DOTM) technique was applied for in-situ fouling observation that allowed for imaging in real time and the determination of the critical flux. FESEM images were captured in order to assess the impact of particle sphericity on the cake structure.

Among PS of different surface charges, the anionic carboxylated PS particles gave the highest critical flux values, but the difference decreased at higher CFV. Regarding particle sphericity, non-spherical particles gave higher critical flux values, with the lower-sphericity peanut-shaped PS out-performing the pear-shaped one. This is tied to the looser cake formed by non-spherical particles due to their varied orientations. The relative difference among PS of different sphericities is maintained at higher CFV, unlike that for PS of different surface charges.

The shear-induced diffusion model is able to provide reasonable predictions despite the variations of the particle charge and shape. The DLVO and XDLVO calculations indicate that the unmodified PS had the highest interaction energy with the membrane surface, which explains the highest fouling performance. Unlike the DLVO calculations, the XDLVO calculations gave a much better agreement with the *J_crit_* trends of both spherical and non-spherical PS particles. As for the flux decline trends, both the DLVO and XDLVO results are in good agreement.

## Figures and Tables

**Table 1:** The surface tension components of the alumina membrane, water, and polystyrene (PS) were found from earlier reports.

	$\gamma^{TOT}\;(\mathrm{mJ}/m^{2})$	$\gamma^{LW}\;(\mathrm{mJ}/m^{2})$	$\gamma^{AB}\;(\mathrm{mJ}/m^{2})$	$\gamma^{+}\;(\mathrm{mJ}/m^{2})$	$\gamma^{-}\;(\mathrm{mJ}/m^{2})$	Ref.
Al_2_O_3_ (Membrane)	39.6	31.6	8.0	0.6	27.2	[35]
Water	72.8	21.8	51.0	25.5	25.5	[36]
Polystyrene (PS)	42.0	42.0	0	0	1.1	[35]

**Table 2:** Characteristics of the different PS microparticles.

PS Type	Vendor-Given Particle Dimensions (µm)	Measured Particle Dimensions via FESEM (µm)	Measured Mean Particle Sizes via Particle Analyzer (µm)	Particle Sphericity	Surface Group	Surfactant	Zeta Potential (mV)
Unmodified	5.1 ± 0.16	5.1 ± 0.10	2.8 ± 0.39	1.00	Sulfate	Anionic	−53.23 ± 0.08
Carboxylated	5.2 ± 0.18	5.1 ± 0.06	2.2 ± 0.31	1.00	Carboxyl	Anionic	−16.28 ± 0.45
Aminated	5.3 ± 0.16	5.3 ± 0.09	2.4 ± 0.23	1.00	Amino	Cationic	15.62 ± 4.65
Pear-shaped	3.8 by 5.1	3.9 by 5.4	1.4 ± 0.38	0.82	Sulfate	Anionic	−41.41 ± 0.42
Peanut-shaped=	3.4 by 5.1	3.0 by 5.1	1.1 ± 0.18	0.52	Sulfate	Anionic	−44.75 ± 0.23

## Data Availability

Not applicable.
